# Supplementary material for: Transverse orbital angular momentum and polarization entangled spatiotemporal structured light
Source: Nanophotonics. 2025 Feb 13;14(6):863–71. doi: 10.1515/nanoph-2024-0764 (PMC11964134; doi:10.1515/nanoph-2024-0764)
Supplement: Supplementary file 1 — Supplementary Material Details [file j_nanoph-2024-0764_suppl_001.docx]

Supplemental Material for

Transverse orbital angular momentum and polarization entangled spatiotemporal structured light

Hsiao-Chih Huang^1,2^, Kefu Mu^2^, Hui Min Leung^2*^, and Chen-Ting Liao^1*^

^1^Department of Physics, Indiana University, Bloomington, Indiana 47405, U.S.A.

^2^Department of Intelligent Systems Engineering, Indiana University, Bloomington, Indiana 47405, U.S.A

**Section 1.** **Theory of the superpositions of t-OAM modes**

The ideal form of the wave function of line-STOVs with a spatiotemporal topological charge *q* can be expressed as a function in cylindrical coordinates, $\psi\left( \rho,\Phi\right)$, with the radial coordinate, $\rho\left( x,t \right)=\rho(x,z')=\sqrt{x^{2}+({z^{'})}^{2}}$, and azimuthal coordinate, $\Phi\left( x,t \right)=\Phi(x,z')={tan}^{-1} \left( x/z' \right)$*.* Here, $z^{'}=z-v_{g}t$ represents the space-like coordinate in the moving reference frame of a pulsed beam travelling with group velocity $v_{g}$. The wave function of t-OAM of light reads

$\psi\left( \rho,\Phi\right)=\left\langle\rho,\Phi|\psi\left( \rho,\Phi\right) \right\rangle=f\left( \rho\right)e^{iq\Phi}$, (1)

where $\left| \psi\left( \rho,\Phi\right) \right\rangle$ is the quantum state of t-OAM of a photon, denotated by Dirac’s *ket* notation and $f\left( \rho\right)$ is a function of the beam’s parameters. The expectation value of energy of our t-OAM of light can be formulated within the framework of quantum mechanics using the photon energy operator $\hat{H}$ as:

$\left\langle E \right\rangle=\frac{\int_{0}^{a} \int_{0}^{2\pi} \hat{H}\left| \psi\left( \rho,\Phi\right) \right|^{2}d\Phi d\rho}{\int_{0}^{a} d\rho\int_{0}^{2\pi} d\Phi}=\frac{1}{2\pi a}\int_{0}^{a} \int_{0}^{2\pi} \hat{H}\left| \psi\left( \rho,\Phi\right) \right|^{2}d\Phi d\rho,$ (2)

where *a* is the beam radius and the operator is defined as $\hat{H}=n\hbar\omega\hat{I},$ where n is the photon number (n = 0, 1, 2, …), $\hat{I}$ is the identity operator, and ω is the central frequency of the light beam. Therefore, we can rewrite the expectation value of energy as:

$\left\langle E \right\rangle=\frac{1}{2\pi a}\int_{0}^{a} \int_{0}^{2\pi} n\hbar\omega\hat{I}\left| \psi\left( \rho,\Phi\right) \right|^{2}d\Phi d\rho=n\hbar\omega A,$ (3)

where $A=\frac{1}{a}\int_{0}^{a} \left| f\left( \rho\right) \right|^{2}d\rho$ is a constant derived from the radial variable related to the light beam used. Now we consider averaged power from our pulsed light beam, $\left\langle P \right\rangle=\left\langle E \right\rangle/T=n\hbar\omega A/T$, where *T* is our nominal data acqusition time (averaging time) and $T\gg t_{0}\gg\frac{2\pi}{\omega}$, $t_{0}$ is the nominal pulse duration (~200 fs) and $\frac{2\pi}{\omega}$ is one optical cycle (~3.4 fs) . When taking n = 1, we get the averaged, single-photon level expression of our t-OAM of light.

A light beam created by the superposition of two t-OAM light ($\left| \psi_{1} \right\rangle$ and $\left| \psi_{2} \right\rangle$) with topological charges $q_{1}$ and $q_{2}$, respectively, with relative phase, *δ*, is expressed in a normalized quantum state as:

$\left| \psi\left( \rho,\Phi\right) \right\rangle=\frac{\left| \psi_{1} \right\rangle+e^{i\delta}\left| \psi_{2} \right\rangle}{\sqrt{\left\langle\psi_{1} | \psi_{1} \right\rangle+\left( e^{-i\delta}\left\langle\psi_{2} \right| \right)\left( e^{i\delta}\left| \psi_{2} \right\rangle\right)}}=\frac{1}{\sqrt{2}\left| f\left( \rho\right) \right|}\left( \left| \psi_{1} \right\rangle+e^{i\delta}\left| \psi_{2} \right\rangle\right).$ (4)

The radial function $f\left( \rho\right)$ is assumed to be identical for those of the two t-OAM light, while the azimuthal function $e^{iq_{1}\Phi}$ and $e^{iq_{2}\Phi}$ correspond to the usage in Eq. (1). Substituting Eq. (4) into Eq. (2) gives:

$$\left\langle P \right\rangle\text{ }=\frac{1}{T}\frac{1}{2}\left( \frac{1}{2\pi a} \right)[\int_{0}^{a} d\rho\int_{0}^{2\pi} d\Phi\left\langle\psi_{1}|\rho,\Phi\right\rangle\left\langle\rho,\Phi\right|\hat{H}\left| \psi_{1} \right\rangle$$

$$+\left\langle\psi_{2}|\rho,\Phi\right\rangle\left\langle\rho,\Phi\right|\hat{H}\left| \psi_{2} \right\rangle$$

$$+e^{i\delta}\left\langle\psi_{1}|\rho,\Phi\right\rangle\left\langle\rho,\Phi\right|\hat{H}|\left. \psi_{2} \right\rangle$$

$\left. +e^{-i\delta}\left\langle\psi_{2}|\rho,\Phi\right\rangle\left\langle\rho,\Phi\right|\hat{H}|\left. \psi_{1} \right\rangle\right]$

$=\frac{n\hbar\omega A}{2T}\left[ 1+1+\frac{e^{i\delta}}{2\pi}\int_{0}^{2\pi} e^{i\left( q_{2}-q_{1} \right)\Phi}d\Phi\right.\left. +\frac{e^{-i\delta}}{2\pi}\int_{0}^{2\pi} e^{-i\left( q_{2}-q_{1} \right)\Phi}d\Phi\right].$ (5)

The two cross terms in the last two integrals of Eq. (5) evaluate to be 2π when $q_{1}\text{= }q_{2}$ and zero when $q_{1}\neq q_{2}$. For the superposition of two beams with identical t-OAM ($q_{1}=q_{2}$), the averaged power varies as

$\left\langle P \right\rangle\text{ = }n\hbar\omega A\left( 1+\cos\delta\right)/T$. (6)

Therefore, the maximum power is $P_{max}=2n\hbar\omega A/T$, and the minimum power, $P_{min}=0,$occur when the superposition is in-phase (*δ* = 0) and out-of-phase (*δ* = π), respectively. In contrast, for the superposition of different t-OAM ($q_{1}\neq q_{2}$), the power remains constant, $P=n\hbar\omega A/T$, regardless of *δ.*

Visibility, $V$, which is defined by the mean power integrated across the beam, is expressed as

$V=|P_{max}-P_{min}|/(P_{max}+P_{min})$. (7)

Therefore, we conclude that when $q_{1}=q_{2}$​ and when $q_{1}\neq q_{2}$, the visibility is 1 and 0, respectively. Equation (7) is what we used in our experiments and mentioned in the main manuscript.

**Section 2. Experimentally measured intensity profiles on the x-y plane for identical and orthogonal t-OAM.**

*
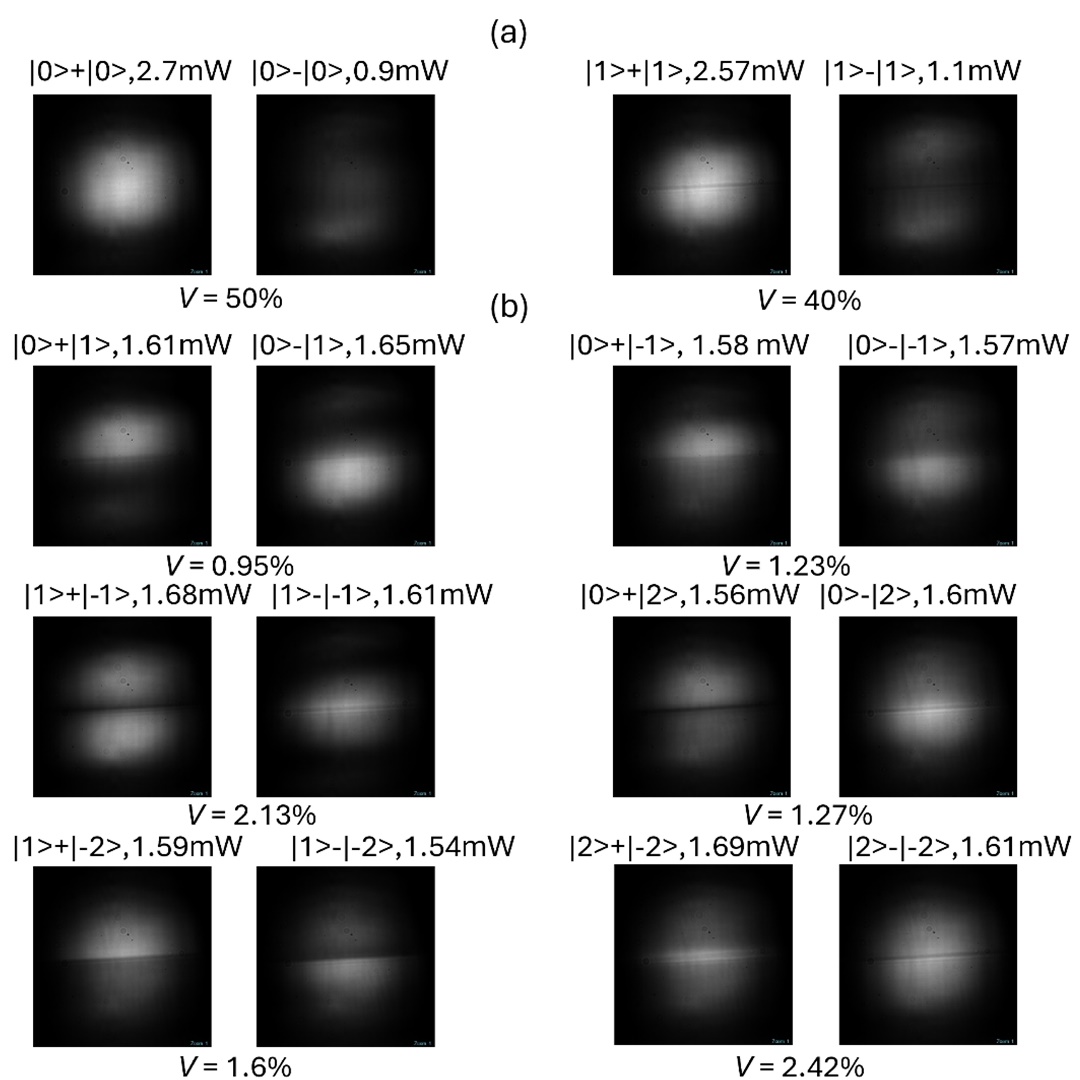
*

Figure S1. Experimentally measured intensity profiles on the x-y plane for the superpositions between (a) identical t-OAM with |0⟩±|0⟩ and |1⟩±|1⟩ and (b), different t-OAM with |0⟩±|1⟩, |0⟩±|-1⟩, |1⟩±|-1⟩, $\left. |0 \right\rangle\pm\left. |2 \right\rangle$, |1⟩±|-2⟩, and |2⟩±|-2⟩.

**Section 3. Schematic experimental setup for generating and analyzing mode entangled spatiotemporal structured light.**

The experimental setup for generating and analyzing mode entanglement is shown in Figure S2. This setup consists of a mode entanglement subsystem, a reference subsystem, a polarization analyzer, an imaging spectrometer, and a beam profiler.

Mode entanglement subsystem: The mode entanglement subsystem is similar to that depicted in Fig. 2(a), but with two key distinctions. Firstly, a thin plate polarizing beamsplitter (PBS1) replaces the thin plate nonpolarizing beamsplitter. Secondly, a half-wave plate (HWP1) angled at 22.5° is added to the system. These modifications enable the mode entanglement of t-OAM of *q*_1_ = 1 and off-diagonal polarization (A1) with t-OAM of *q*_2_ = -1 and diagonal polarization (D2), which can be expressed as |Ψ⟩ = |q=1⟩|A⟩ ± |q=-1⟩|D⟩. We note that, compared to the optical path toward SLM2, the path toward SLM1 includes an additional reflection from PBS1. Therefore, the phase pattern on SLM2 must be adjusted accordingly to compensate for the resulting OAM helicity change. The pair of orthogonal linear polarizations of A1 and D2 were subsequently converted to another pair of orthogonal circular polarizations, R1 and L2, after passing through a quarter-wave plate (QWP1) angled at 0°. At this point, we have generated our targeted mode entangled light, expressed as |Ψ⟩=|+1⟩|R⟩±|-1⟩|L⟩. This light is then directed towards three optical characterization subsystems, namely the polarization analyzer, imaging spectrometer, and beam profiler.

Polarization analyzer: The polarization analyzer was used to verify the polarization states generated by the mode entanglement subsystem described above. To aid the polarization analysis, the orthogonal pair of circular polarizations were first converted into linear polarizations (S1 and P2) through the use of a quarter-wave plate (QWP2) angled as 45°. The light was then sent through a rotatable half-wave plate and linear polarizer (LP) before the time-averaged optical power of the entire beam was captured by a power meter (P).

Imaging spectrometer: The t-OAM charges |+1⟩ and |-1⟩ can be distinguished by an imaging spectrometer as follows. First, two beams pass through a cylindrical lens (CL2v) with a focal length of f=70 cm. The beams are diffracted by a thin plate transmission grating (G2) with a groove density of 1600 lines/mm and then focused by a vertical cylindrical lens (CL3v) with a focal length of f=13 cm. The resultant profiles are captured on a beam profiler camera (C2), which is located at the focal plane of CL2v.

Reference subsystem: It is crucial to introduce a phase shift of either 0 (namely, $e^{i\delta}=e^{i(0)}=e^{i(2\pi)}=+1$) or π (namely, $e^{i\delta}=e^{i(\pi)}=-1$) between |1⟩|R⟩ and |-1⟩|L⟩ to achieve in-phase or out-of-phase superpositions, respectively. Because the imaging spectrometer does not allow these two conditions to be distinguished, a reference subsystem described here was used. The SLM3 in this subsystem is loaded with a phase pattern of LG(0,1) to generate a beam with |1⟩. The first index in the LG pattern is set to zero as we only used the azimuthal index for OAM. When the original s-polarized light is sent into the reference subsystem, it is split into two arms by a beamsplitter (BS3). In one arm, the light is converted into polarization of either A3 or D3 by using a half-wave plate (HWP2) oriented at ±22.5°. After exiting the reference subsystem, these states were subsequently converted to circular polarizations (i.e., R3 or L3) by passing through QWP1 at 0°. This reference beam is then overlapped with the light returning from either SLM1 (i.e., |1⟩|R⟩) or SLM2 (i.e., |-1⟩|L⟩) individually. We achieve in-phase superposition to generate |+1⟩|R⟩+|-1⟩|L⟩ by adjusting the translational stages controlling SLM1 and SLM2 to ensure constructive interference between both the reference and |1⟩|R⟩, as well as the reference and |-1⟩|L⟩. Conversely, the out-of-phase superposition is achieved by introducing an additional π phase delay in either arm associated with |1⟩|R⟩ or |-1⟩|L⟩. This is again accomplished by adjusting the translational stages of either SLM1 or SLM2 to create constructive interference between the reference and |1⟩|R⟩ and destructive interference between the reference and |-1⟩|L⟩.


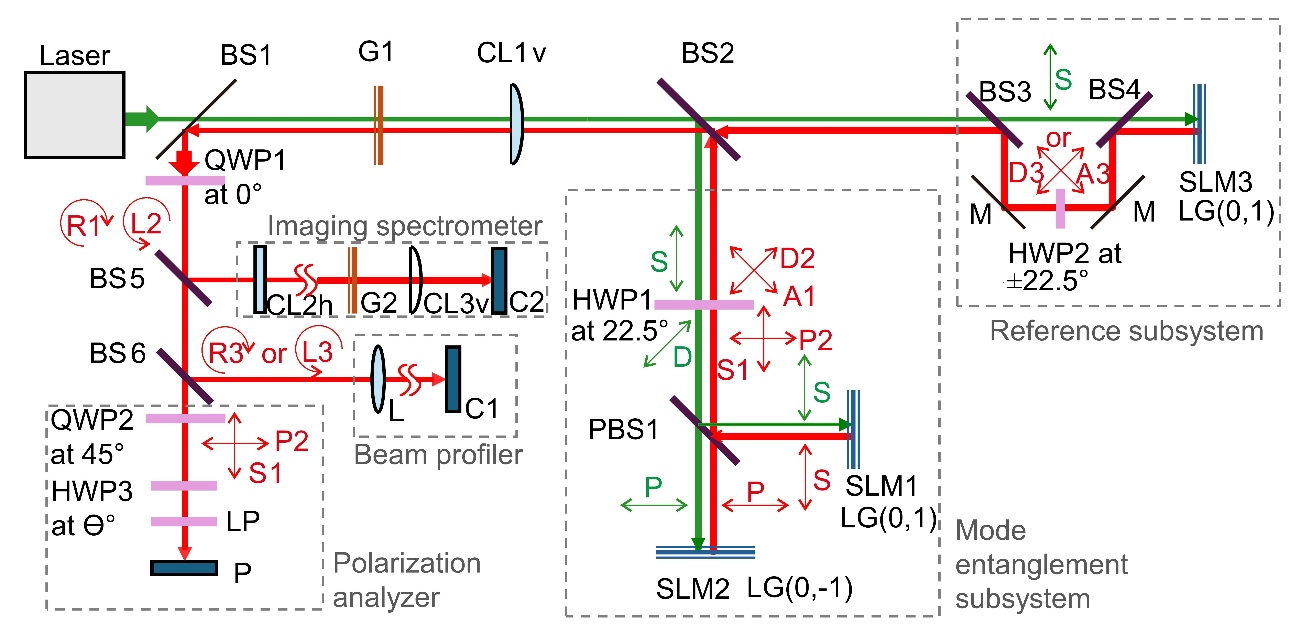


Figure S2. Experimental setup for generating and analyzing the mode entanglement of t-OAM and polarization. This comprises a folded 4*f* pulse shaper and a Michelson interferometer with HWP1 and PBS1, a reference subsystem, an imaging spectrometer, a beam profiler, and a polarization analyzer, indicated by five dashed boxes. BS: thin plate non-polarizing beam splitter; PBS: thin plate polarizing beam splitter; HWP: half-wave plates; QWP: quarter-wave plates; G: transmission grating; CLh and CLv: cylindrical lenses with horizontal and vertical focal lines; L: spherical lens; SLM: spatial light modulator; C: camera; P: power meter.

**Section 4. Theory showing t-OAM and circular polarization mode entangled light are vector beams with spatiotemporal dependent polarization.**

The electric field of a beam carrying t-OAM can be represented as

${E\left( x,y,z,t \right)\propto Ue^{iq\Phi_{ST}(x,t)}e}^{i(k_{z}z-\omega t)}$= $e^{iq\Phi_{ST}}e^{ik_{z}(z-v_{g}t)}$

It propagates along the z-axis, where $z^{'}=z-v_{g}t$ represents space-like coordinate in the moving reference frame of a laser pulse travelling with group velocity $v_{g}$, where $v_{g}\sim c$.

Neglecting the carrier term$e^{i(k_{z}z-\omega t)}$, the main feature of t-OAM stems from its spatiotemporal phase term that reads

$e^{iq\Phi_{ST}\left( x,t \right)}=e^{iq\Phi_{ST}\left( x,z^{'} \right)}$= $[cos{(q\Phi}_{ST})]\pm i[sin({q\Phi}_{ST})]$, where$\text{ }\Phi_{ST}={tan}^{-1}(\frac{x/x_{0}}{z^{'}/\eta z_{0}^{'}})$.

In the discussion below, we drop the subscript spatiotemporal and use $\Phi=\Phi_{ST}(x,z')$ for simplicity. We also assume $x_{0}$= $z_{0}^{'}=1$ and $\eta=1$ for a simplified case.

Right-hand circularly polarized (R) light and left-hand circularly polarized (L) light can be represented as

$\left\{ \begin{aligned} &\left| R(x,y) \right\rangle\\ &\left| L(x,y) \right\rangle\end{aligned} \right.\propto\left| P(x,y) \right\rangle\pm i\left| S(x,y) \right\rangle\propto\left| H(x,y) \right\rangle\pm i\left| V(x,y) \right\rangle$,

where $\left| P \right\rangle, \left| S \right\rangle, \left| V \right\rangle, \left| H \right\rangle$ are p-, s-, vertical-, horizontal- polarized light, respectively.

Here, $\left| P \right\rangle$=$\left| H \right\rangle$ is defined along the y-axis, and $\left| S \right\rangle$=$\left| V \right\rangle$ is defined along the x-axis

Similarity, diagonal and anti-diagonal polarized light can be represented as

$\left\{ \begin{aligned} &\left| D(x,y) \right\rangle\\ &\left| A(x,y) \right\rangle\end{aligned} \right.\propto\left| P(x,y) \right\rangle\pm\left| S(x,y) \right\rangle$

The polarization depends on the position of beam profile in the x-z’ plane for the in-phase and out-of-phase mode entangled light, $\left. |\Psi(x,y,z') \right\rangle=\left| q=1 \right\rangle\left| R(x,y) \right\rangle\pm\left| q=-1 \right\rangle\left| L(x,y) \right\rangle$.

For a mode entangled light with first term and second term in-phase, we can write it as

$$\left. |\Psi(x,y,z') \right\rangle=\left| 1 \right\rangle\left| R\left( x,y \right) \right\rangle+\left| -1 \right\rangle\left| L\left( x,y \right) \right\rangle$$

$$\propto e^{iq\Phi(x,z')}\left| R(x,y) \right\rangle+e^{-iq\Phi(x,z')}\left| L(x,y) \right\rangle$$

Considering the simplest case when $q=1$,

If $\Phi(x,z')=0$ [twelve o’clock orientation at the x-z’ plane],

then we get $\left. |\Psi(x,y,z') \right\rangle=\left| R(x,y) \right\rangle+\left| L(x,y) \right\rangle\propto\left| P(x,y) \right\rangle$

If $\Phi=\frac{\pi}{2}$ [three o’clock orientation at the x-z’ plane],

then we get $\left. |\Psi(x,y,z') \right\rangle=i\left( \left| R(x,y) \right\rangle-\left| L(x,y) \right\rangle\right)\propto-\left| S(x,y) \right\rangle$

If $\Phi=\pi,$ then we get $\left. |\Psi\right\rangle= -\left( \left| R \right\rangle+\left| L \right\rangle\right)\propto-\left| P \right\rangle$

If $\Phi=\frac{3\pi}{2},$ we get $\left. |\Psi\right\rangle= -i\left( \left| R \right\rangle-\left| L \right\rangle\right)\propto\left| S \right\rangle$

If $\Phi=\pm\frac{\pi}{4}$, then we get

$\left. |\Psi\right\rangle= e^{i\Phi}\left| R \right\rangle+e^{-i\Phi}\left| L \right\rangle\propto\left( 1\pm i \right)\left( \left| P \right\rangle+i\left| S \right\rangle\right)+\left( 1\mp i \right)\left( \left| P \right\rangle-i\left| S \right\rangle\right)$

$$=\left[ \left( \left| P \right\rangle\mp\left| S \right\rangle\right)\pm i\left( \left| P \right\rangle\pm\left| S \right\rangle\right) \right]+\left[ \left( \left| P \right\rangle\mp\left| S \right\rangle\right)\mp i\left( \left| P \right\rangle\pm\left| S \right\rangle\right) \right]$$

$$\propto\left| P \right\rangle\mp\left| S \right\rangle$$

$$\propto\left\{ \begin{aligned} &\left| A(x,y) \right\rangle\\ &\left| D(x,y) \right\rangle\end{aligned} \right.$$

If $\Phi=\pm\frac{3\pi}{4}$, then we get

$$\left. |\Psi\right\rangle=e^{i\Phi}\left| R \right\rangle+e^{-i\Phi}\left| L \right\rangle\propto\left( -1\pm i \right)\left( \left| P \right\rangle+i\left| S \right\rangle\right)+\left( -1\mp i \right)\left( \left| P \right\rangle-i\left| S \right\rangle\right)$$

$$=\left[ -\left( \left| P \right\rangle\pm\left| S \right\rangle\right)\pm i\left( \left| P \right\rangle\mp\left| S \right\rangle\right) \right]+\left[ -\left( \left| P \right\rangle\pm\left| S \right\rangle\right)\mp i\left( \left| P \right\rangle\mp\left| S \right\rangle\right) \right]$$

$$\propto-\left( \left| P \right\rangle\pm\left| S \right\rangle\right)$$

$\propto\left\{ \begin{aligned} &-\left| D(x,y) \right\rangle\\ &-\left| A(x,y) \right\rangle\end{aligned} \right.$

For a mode entangled light with first term and second term out-of-phase, we can write it as

$$\left. |\Psi\right\rangle=\left| 1 \right\rangle\left| R \right\rangle-\left| -1 \right\rangle\left| L \right\rangle\sim e^{i\Phi}\left| R \right\rangle-e^{-i\Phi}\left| L \right\rangle$$

If $\Phi=0,$then we get $\left. |\Psi\right\rangle= \left| R \right\rangle-\left| L \right\rangle\propto\left| S \right\rangle$

If $\Phi=\frac{\pi}{2},$then we get $\left. |\Psi\right\rangle= i\left( \left| R \right\rangle+\left| L \right\rangle\right)\propto\left| P \right\rangle$

If $\Phi=\pi, then we get \left. |\Psi\right\rangle=-\left( \left| R \right\rangle-\left| L \right\rangle\right)\propto-\left| S \right\rangle$

If $\Phi=\frac{3\pi}{2},\text{ }\mathrm{then}\text{we get }\left. |\Psi\right\rangle= -i\left( \left| R \right\rangle+\left| L \right\rangle\right)\propto-\left| P \right\rangle$

If $\Phi=\pm\frac{\pi}{4},$ then we get

$${\left. |\Psi\right\rangle=e}^{i\Phi}\left| R \right\rangle-e^{-i\Phi}\left| L \right\rangle\propto\left( 1\pm i \right)\left( \left| P \right\rangle+i\left| S \right\rangle\right)-\left( 1\mp i \right)\left( \left| P \right\rangle-i\left| S \right\rangle\right)=\left[ \left( \left| P \right\rangle\mp\left| S \right\rangle\right)\pm i\left( \left| P \right\rangle\pm\left| S \right\rangle\right) \right]-\left[ \left( \left| P \right\rangle\mp\left| S \right\rangle\right)\mp i\left( \left| P \right\rangle\pm\left| S \right\rangle\right) \right]$$

$$\propto\left| P \right\rangle\pm\left| S \right\rangle\propto\left\{ \begin{aligned} &\left| D \right\rangle\\ &\left| A \right\rangle\end{aligned} \right.$$

If $\Phi=\pm\frac{3\pi}{4},$ then we get

$$\left. |\Psi\right\rangle=\left| R \right\rangle-e^{-i\Phi}\left| L \right\rangle\propto\left( -1\pm i \right)\left( \left| P \right\rangle+i\left| S \right\rangle\right)-\left( -1\mp i \right)\left( \left| P \right\rangle-i\left| S \right\rangle\right)=\left[ -\left( \left| P \right\rangle\pm\left| S \right\rangle\right)\pm i\left( \left| P \right\rangle\mp\left| S \right\rangle\right) \right]-\left[ -\left( \left| P \right\rangle\pm\left| S \right\rangle\right)\mp i\left( \left| P \right\rangle\mp\left| S \right\rangle\right) \right]\propto-\left( \left| P \right\rangle\mp\left| S \right\rangle\right)$$

$$\propto\left\{ \begin{aligned} &-\left| A \right\rangle\\ &-\left| D \right\rangle\end{aligned} \right.$$

We show the local polarization directions in two planes of x-z’ and x-y of two mode entangled spatiotemporal structured lights with |Ψ⟩=|+1⟩|R⟩±|-1⟩|L⟩ in the Figs. 3(a) and 3(c) of the main manuscript. In the x-y plane, the time average polarizations for |+1⟩|R⟩±|-1⟩|L⟩ are all P (parallel to the y-axis) and S (parallel to the x-axis), respectively, and gradually increasing in magnitude along the x-axis from its beam center.
